# A study on inter‐planner plan quality variability using a manual planning‐ or Lightning dose optimizer‐approach for single brain lesions treated with the Gamma Knife^®^ Icon™

**DOI:** 10.1002/acm2.14088

**Published:** 2023-07-06

**Authors:** Yongsook C. Lee, D Jay Wieczorek, Vibha Chaswal, Rupesh Kotecha, Matthew D. Hall, Martin C. Tom, Minesh P. Mehta, Michael W. McDermott, Alonso N. Gutierrez, Ranjini Tolakanahalli

**Affiliations:** ^1^ Department of Radiation Oncology Miami Cancer Institute Baptist Health South Florida Miami USA; ^2^ Department of Radiation Oncology Herbert Wertheim College of Medicine Florida International University Miami USA; ^3^ Department of Translational Medicine Herbert Wertheim College of Medicine Florida International University Miami USA; ^4^ Department of Neurosurgery Miami Neuroscience Institute Baptist Health South Florida Miami USA

**Keywords:** brain metastasis, fast inverse planning (FIP), Gamma Knife^®^ Icon™, Lightning, manual forward planning, post‐operative resection cavity, stereotactic radiosurgery, stereotactic radiotherapy, vestibular schwannoma

## Abstract

**Purpose:**

The purpose of this study is to investigate inter‐planner plan quality variability using a manual forward planning (MFP)‐ or fast inverse planning (FIP, Lightning)‐approach for single brain lesions treated with the Gamma Knife^®^ (GK) Icon™.

**Methods:**

Thirty patients who were previously treated with GK stereotactic radiosurgery or radiotherapy were selected and divided into three groups (post‐operative resection cavity, intact brain metastasis, and vestibular schwannoma [10 patients per group]). Clinical plans for the 30 patients were generated by multiple planners using FIP only (1), a combination of FIP and MFP (12), and MFP only (17). Three planners (Senior, Junior, and Novice) with varying experience levels re‐planned the 30 patients using MFP and FIP (two plans per patient) with planning time limit of 60 min. Statistical analysis was performed to compare plan quality metrics (Paddick conformity index, gradient index, number of shots, prescription isodose line, target coverage, beam‐on‐time (BOT), and organs‐at‐risk doses) of MFP or FIP plans among three planners and to compare plan quality metrics between each planner's MFP/FIP plans and clinical plans. Variability in FIP parameter settings (BOT, low dose, and target max dose) and in planning time among the planners was also evaluated.

**Results:**

Variations in plan quality metrics of FIP plans among three planners were smaller than those of MFP plans for all three groups. Junior's MFP plans were the most comparable to the clinical plans, whereas Senior's and Novice's MFP plans were superior and inferior, respectively. All three planners’ FIP plans were comparable or superior to the clinical plans. Differences in FIP parameter settings among the planners were observed. Planning time was shorter and variations in planning time among the planners were smaller for FIP plans in all three groups.

**Conclusions:**

The FIP approach is less planner dependent and more time‐honored than the MFP approach.

## 1 INTRODUCTION

Leksell Gamma Knife^®^ (GK) (Elekta Instrument AB, Stockholm, Sweden) is a dedicated intracranial stereotactic radiosurgery (SRS) system used for the management of malignant and benign tumors as well as functional and vascular disorders.^1^, ^2^, ^3^ The Icon™ platform, a new generation of a GK unit, incorporates kilovoltage cone beam computed tomography for the definition of a stereotactic reference frame and daily volumetric image guidance as well as additional frameless system with intra‐fractional motion management.^4^ With the advent of the Icon platform, stereotactic radiotherapy (SRT) with treatment in three or more fractions has now become feasible on the GK unit.^5^, ^6^ Like the Perfexion™, the Icon houses 192 sealed cobalt‐60 sources arranged in eight motorized sectors, each containing 24 sources.^4^, ^7^ Each sector is controlled independently and can be in one of four collimator states: 4, 8, 16 mm, or beam‐off (block) state.^8^ In each GK shot, the radiation is delivered for a specified time at an isocenter position with a given collimator configuration composed of various sector settings.^1^, ^9^

Traditionally, GK planning has been performed in a manual planning approach. In this approach, the planner manually places shots within the target and iteratively adjusts their locations, collimator configurations, and relative weights.^10^ Given the high degree‐of‐freedom in the dose distribution (e.g., 65 536 possible beam shapes per shot),^8^ it is not possible to explore all potential options using this approach.^1^ Additionally, manual planning is time‐consuming and plan quality heavily relies on the planner's experience and planning time invested.^10^

Elekta AB introduced inverse planning (IP) tools in the Leksell GammaPlan^®^ (LGP) (Elekta Instrument AB, Stockholm, Sweden). The first IP tool released in 2010 uses well‐established metrics such as target coverage (TC), selectivity, and gradient index (GI) at a predetermined prescription isodose level with a beam‐on time (BOT) penalization to determine isocenter positions, collimator configurations, and beam weights.^1^ However, this IP tool has difficulty in obtaining an optimal solution due to the use of relative isodoses and the variability of the shot positions.^1^ In 2020, Elekta AB released a new IP dose optimizer, called fast inverse planning (FIP) and, commercially referred to as Lightning. This optimizer uses a linear objective function for sector duration optimization^1^ and has been shown to generate plans quickly with none or minimal manual adjustments.^8^

There are a few published studies including the vendor's initial study comparatively evaluating FIP (Lightning) versus clinical manual planning for selected brain lesions.^1^, ^5^, ^8^, ^9^ However, Sjölund et al. and Wieczorek et al. did not investigate inter‐planner variability when employing FIP.^1^, ^8^ Cui et al. included three planners for FIP plans but their study was primarily focused on the comparison between FIP and clinical manual planning.^9^ Spaniol et al. assessed inter‐planner variability in both manual planning and FIP, but planning was performed by three experienced planners and the variability among the planners was evaluated only for three patients with different diagnosis.^5^ Moreover, none of the studies were “real‐world”, in other words, limiting planning time, which is a necessity in clinical practice. Knowing this, we investigated inter‐planner plan quality variability in manual forward planning (MFP) and FIP performed with a realistically pre‐fixed planning time limit of 60 min for single brain lesions treated with a GK Icon platform. For this study, three planners with varying planning experience levels participated in the evaluation.

## 2 METHODS

### 2.1 Patient cohort

**FIGURE 1:**
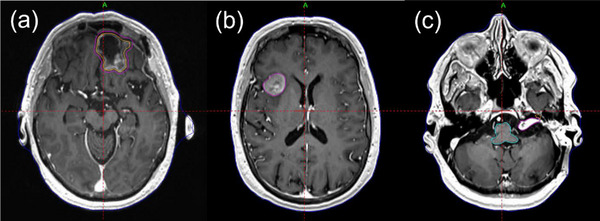
Axial T1 post‐contrast magnetic resonance images demonstrating representative examples from the groups evaluated in this treatment planning study: (a) a post‐operative resection cavity of a brain metastasis, (b) an intact brain metastasis, and (c) vestibular schwannoma

Thirty patients (*n* = 30) who were previously treated with GK SRS or SRT were selected and were divided into three groups (10 patients per group). Group selection was based on the size and shape of the lesions (Table 1). The first group (BM_postop_ group) consisted of post‐operative resection cavities of a brain metastasis and received SRT to a planning target volume (PTV) with a regimen of 30 Gy in five fractions using institutional dosing guidelines.^11^ The clinical target volume was derived from a uniform expansion of 2 mm from the gross target volume (GTV). No setup margin was used to derive the PTV. The second group (BM_intact_ group) had a single, intact brain metastasis and received staged SRS to the GTV to 15 Gy in one fraction per stage based on our institutional paradigm.^12^ For this exercise, the first of the 2‐stage treatment was chosen. The third group (VS group) consisted of vestibular schwannomas (VS) and received SRS to the GTV to 12.5 or 13 Gy in one fraction. Examples of representative target volumes from each of the groups are shown in Figure 1. The lesions in the BM_postop_ and BM_intact_ groups were not adjacent (> 5 cm) to organs‐at‐risk (OARs) such as brainstem and optic chiasm, whereas those in the VS group were adjacent to cochlea and brainstem (Figure 1). Clinical plans for 13 patients (six from the BM_postop_ group, six from the BM_intact_ group, and one from the VS group) of the 30 patients were generated using FIP only or a combination of FIP and MFP, and the rest using MFP. A mask was used for all 10 patients in the BM_post_ group, whereas a frame was used for the entire VS group. For the BM_intact_ group, six patients used a mask, and four patients used a frame for immobilization. For the BM_intact_ group, the selection of the fixation configuration was based on BOT and patient preference. Table 1 shows lesion characteristics, number of patients (lesions), mean volume of the lesions and prescription dose for each group. This study was approved by our Institutional Review Board.

**TABLE 1:** Lesion characteristics, number of patients (lesions), mean volume of lesions, and prescription dose for patient cohort selected in this study.

Lesion characteristics			No. of patients (lesions)	Mean volume of lesions (range) (cc)	Prescription dose
BM_postop_ group	Post‐operative resection cavity of a brain metastasis	Relatively large, irregular shape	10	27.79 (20.22–32.01)	30 Gy in five fractions to PTV
BM_intact_ group	Intact brain metastasis	Medium‐sized, spherical shape	10	5.64 (3.00–9.60)	15 Gy in one fraction to GTV
VS group	Vestibular schwannoma	Relatively small, irregular shape	10	1.17 (0.29–2.04)	12.5 or 13 Gy in one fraction to GTV

Abbreviations: GTV, gross tumor volume; PTV, planning target volume.

### 2.2 Treatment planning

Three planners (Senior [experience > 5 years], Junior [experience < 2 years], and Novice [experience < 3 months]) replanned the 30 clinical cases based on our institutional GK planning criteria. Each planner generated two plans (one plan using MFP and the other plan using FIP) per case, totaling 60 plans. Planning time was limited to 60 min for both MFP and FIP plans. Our institutional GK planning criteria include OAR doses < given constraints, TC ≥ 99.5% receiving a prescription dose or higher, Paddick conformity index (PCI) as close to 1.0 as possible, GI ≤ 3.0 and a prescription isodose line (IDL) ≥ 50%. For the VS group, cochlea mean dose from each clinical plan was provided to the planners as a guided tolerance. A maximum point dose of 12.5 Gy for uninvolved brainstem was also given.^13^ For the clinical plans, a cochlea mean dose of 6 Gy was used as the OAR constraint.^14^, ^15^ When cochlea abutted the GTV, planners planned for mean dose as low as reasonably achievable while not compromising TC. MFP and FIP were performed as follows.

MFP plans were generated with or without the assistance of the IP tool in LGP (v11.3.2). First, a prescription dose was entered and prescription IDL > 50% was selected based on the size of the lesion. Second, shots were placed using the Shot Fill technique under the IP settings. Either a single collimator size or composite shots were used. If necessary, shot(s) were manually placed to minimize OAR doses (for the VS group). Third, shot isocenter positions, sector configurations and weights were optimized using the IP optimization tool followed by manual adjustments. Under the IP settings, relative importance settings ranging from 0.00 to 1.00 for Coverage, Selectivity (= PCI/Coverage)^1^, GI, and Beam‐on were selected and the IP optimizer was run repeatedly with different relative importance settings until TC, PCI, BOT, and GI were achieved in the order of this importance. Then manual fine adjustments were made until the time limit to improve OAR dose(s) (for the VS group), TC (at least 99.5%) and other plan quality. When the IP optimizer was not used, shot isocenter position, sector configurations, and weights were manually adjusted until the time limit or until the OAR dose(s) (for the VS group), TC (at least 99.5%), PCI, BOT, and GI were achieved in the order of importance. Number of iterations was defined as number of times the IP optimizer was run or manual planning without the IP optimizer was repeated from scratch.

FIP plans were generated using the Lightning dose optimizer of LGP (v11.3.2). A prescription dose, max dose to the target, and number of fractions (one or five, only for mask fixation configuration) were entered and the TC box was enabled. Max dose to the target was selected by multiplying a prescription dose by 1.8–1.9 to achieve prescription IDL ≥ 50%. Then low dose (LD) and BOT settings ranging from 0.00 to 1.00 each were selected and the FIP optimizer was run repeatedly with different LD/BOT combinations until the OAR doses (for the VS group), TC (at least 99.5%), PCI, BOT, and GI were achieved in the order of this importance. Number of iterations was defined as number of times the FIP optimizer was run. FIP parameters were set such that prescription IDL was not lower than 50%. To minimize planner dependence on planning, no manual adjustments were made after the FIP optimizer was run. For the VS group, to achieve mean dose comparable to that from the clinical plans, the planners entered adequate max dose to cochlea under risk zones of the FIP setting.

### 2.3 Comparison of plan quality metrics among three planners

The following plan quality metrics of MFP or FIP plans were noted by three planners: OAR doses (Gy) (for the VS group), TC (%), PCI, sBOT (scaled BOT) (min), GI, prescription IDL (%), number of shots and planning time (min). TC up to one decimal point (%) was taken from Statistics of Dose Evaluation in LGP (v.11.3.2). PCI and GI are defined in Paddick's studies.^16^, ^17^ sBOT was defined as BOT scaled to a dose rate of 3.0 Gy/min. In addition, selected final LD and BOT settings as well as entered target max dose for FIP plans were noted.

Statistical analysis was performed (i) to compare plan quality metrics of MFP or FIP plans among three planners and (ii) to compare plan quality metrics between each planner's MFP/FIP plans and the clinical plans. A normality test was performed first. When plan quality metrics from all three planners passed a normality test, a one‐way ANOVA test was performed to compare plan quality metrics of MFP or FIP plans among three planners. Otherwise, a Kruskal–Wallis test was performed. Similarly, for normal distributions, a paired *t*‐test (two tailed) was performed to compare plan quality metrics between each planner's plans and the clinical plans. Otherwise, a Wilcoxon matched pairs test was performed. A *p*‐value < 0.05 was considered statistically significant.

## 3 RESULTS

### 3.1 Plan quality metrics

**FIGURE 2:**
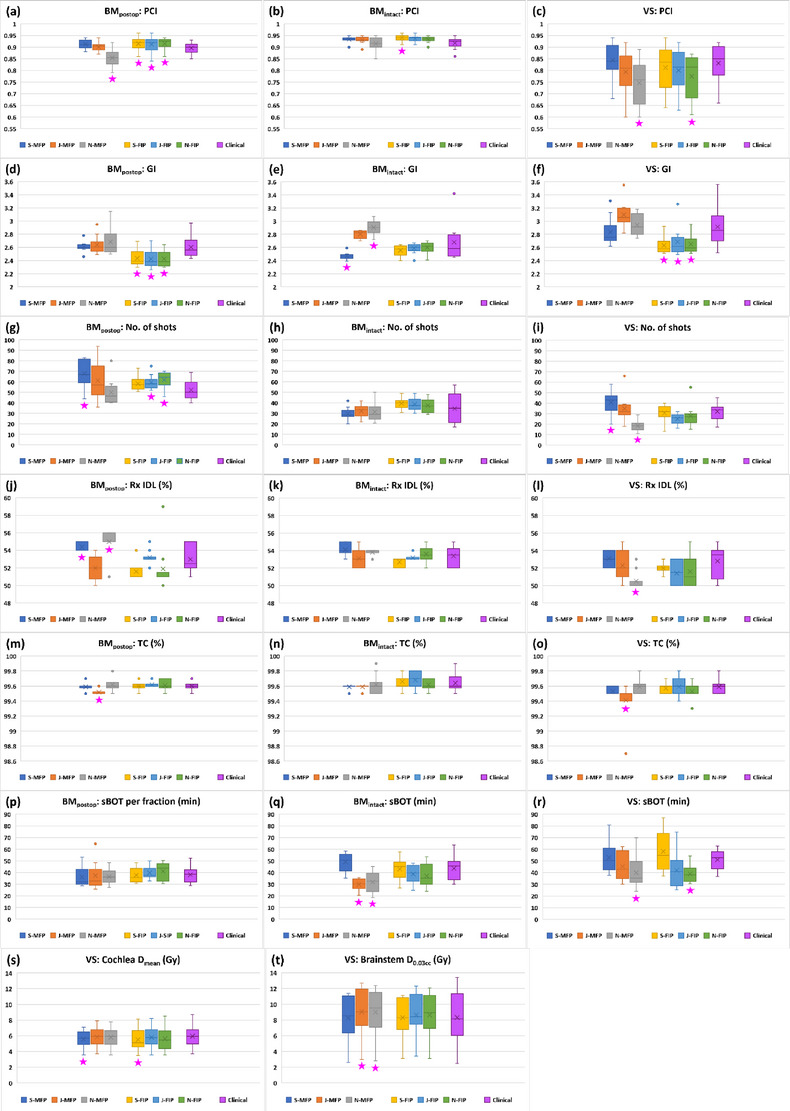
Comparison of plan quality metrics of MFP or FIP plans among three planners. Clinical plans are also plotted. Pink stars represent the MFP or FIP plans whose plan quality metrics are statistically significantly different from those of the clinical plans. Abbreviations: BM_intact_, intact brain metastasis; BM_postop_, post‐operative resection cavity of a brain metastasis; FIP, fast inverse planning; GI, gradient index; J, Junior; MFP, manual forward planning; N: Novice; PCI, Paddick conformity index; Rx IDL, prescription isodose line; S, Senior; sBOT, scaled beam‐on time; TC, target coverage; VS, vestibular schwannoma.

Figure 2 shows the comparison of plan quality metrics (PCI [a–c], GI [d–f], number of shots [g–i], prescription IDL [j–l], TC [m–o], sBOT [p–r], and OAR doses [s–t]) of MFP or FIP plans among the three planners for all three groups. Clinical plans are also plotted. In each figure, Box and Whisker plots from left to right represent Senior's MFP (S‐MFP), Junior's MFP (J‐MFP), Novice's MFP (N‐MFP), Senior's FIP (S‐FIP), Junior's FIP (J‐FIP), Novice's FIP (N‐FIP), and clinical plans in order. Each plot displays lower and upper quartiles (box), median (horizontal line inside the box), mean (marker × inside the box), minimum and maximum values (two lines outside the box) and outlier(s) (dot[s]) of data.

Table 2 presents the mean values of maximum differences in plan quality metrics of MFP or FIP plans among the three planners and the results of statistical analysis. The mean values for MFP plans were greater than those for FIP plans in all three groups except TC for the BM_intact_ group and, sBOT and cochlea mean dose for the VS group. For the BM_postop_ group, PCI, number of shots, prescription IDL and TC of MFP plans were statistically significantly different (*p* < 0.05) among the planners, whereas PCI, number of shots, and TC of FIP plans were not (*p* > 0.05). Prescription IDL of both MFP and FIP plans was significantly different (*p* < 0.05) among the planners. For the BM_intact_ group, GI and sBOT of MFP plans were statistically significantly different (*p* < 0.05) among the planners but those of FIP plans were not (*p* > 0.05). Prescription IDL was significantly different (*p* < 0.05) among the planners only for FIP plans. For the VS group, PCI, GI, number of shots, prescription IDL, and TC of MFP plans were statistically significantly different (*p* < 0.05) among the planners, while those of FIP plans all were not (*p* > 0.05). sBOT was significantly different (*p* < 0.05) among the planners only for FIP plans.

**TABLE 2:** Mean values of maximum differences in plan quality metrics of MFP or FIP plans among three planners and the results of a one‐way ANOVA test (parametric) or a Kruskal–Wallis test (non‐parametric) among the planners.

	BM_postop_ group				BM_intact_ group				VS group			
	Mean of max diff. among three planners		ANOVA/Kruskal–Wallis test among three planners		Mean of max diff. among three planners		ANOVA/Kruskal–Wallis test among three planners		Mean of max diff. among three planners		ANOVA/Kruskal–Wallis test among three planners	
	MFP	FIP	MFP	FIP	MFP	FIP	MFP	FIP	MFP	FIP	MFP	FIP
PCI	0.06 ± 0.03	0.01 ± 0.01	*p* < 0.05	*p* = 0.9664	0.02 ± 0.02	0.01 ± 0.01	*p* = 0.1262	*p* = 0.5616	0.10 ± 0.04	0.04 ± 0.05	*p* < 0.05	*p* = 0.6859
	(0.01–0.10)	(0.00–0.03)			(0.00–0.05)	(0.00–0.02)			(0.05–0.18)	(0.00–0.12)		
GI	0.16 ± 0.09	0.04 ± 0.03	*p* = 0.5760	*p* = 0.9793	0.44 ± 0.09	0.06 ± 0.05	*p* < 0.05	*p* = 0.4639	0.31 ± 0.24	0.11 ± 0.09	*p* < 0.05	*p* = 0.7637
	(0.08–0.37)	(0.00–0.10)			(0.34–0.57)	(0.01–0.20)			(0.08–0.93)	(0.03–0.34)		
No. of shots	26.6 ± 15.1 (5–54)	9.8 ± 5.0 (2–17)	*p* < 0.05	*p* = 0.5082	10.4 ± 5.3 (4–17)	4.8 ± 3.3 (1–12)	*p* = 0.7255	*p* = 0.7527	26.2 ± 9.6 (17–46)	9.8 ± 9.8 (2–35)	*p* < 0.05	*p* = 0.3169
Rx IDL (%)	3.6 ± 1.6 (1–6)	2.3 ± 1.1 (1–5)	*p* < 0.05	*p* < 0.05	1.6 ± 0.8 (0–3)	1.2 ± 0.9 (0–2)	*p* = 0.0611	*p* < 0.05	3.0 ± 0.8 (2–4)	1.6 ± 0.8 (1–3)	*p* < 0.05	*p* = 0.5763
TC (%)	0.14 ± 0.10 (0.0–0.3)	0.09 ± 0.07 (0.0–0.2)	*p* < 0.05	*p* = 0.7909	0.09 ± 0.12 (0.0–0.3)	0.17 ± 0.07 (0.1–0.3)	*p* = 0.9929	*p* = 0.3266	0.21 ± 0.26 (0.0–0.9)	0.13 ± 0.08 (0.0–0.3)	*p* < 0.05	*p* = 0.4069
sBOT (min)	11.6 ± 7.5 (5.1–28.8)	7.2 ± 5.0 (1.6–16.9)	*p* = 0.9504	*p* = 0.5283	20.5 ± 4.1 (15.2–26.9)	7.6 ± 5.6 (2.0–19.9)	*p* < 0.05	*p* = 0.3328	17.0 ± 10.4 (5.6–33.4)	24.2 ± 11.7 (7.2–47.0)	*p* = 0.1130	*p* < 0.05
Cochlea D_mean_ (Gy)	N/A	N/A	N/A	N/A	N/A	N/A	N/A	N/A	0.3 ± 0.2 (0.1–0.8)	0.4 ± 0.4 (0.1–1.2)	*p* = 0.8913	*p* = 0.9031
Brainstem D_0.03cc_ (Gy)	N/A	N/A	N/A	N/A	N/A	N/A	N/A	N/A	1.0 ± 0.5 (0.4–1.7)	0.6 ± 0.4 (0.1–1.2)	*p* = 0.7847	*p* = 0.9477
Planning time (min)	15.7 ± 9.6 (1–32)	9.4 ± 4.7 (2–17)	*p* < 0.05	*p* = 0.0749	21.6 ± 14.8 (2–46)	2.2 ± 1.4 (0–4)	*p* < 0.05	*p* = 0.1612	32.9 ± 9.8 (19–50)	15.1 ± 9.8 (3–31)	*p* < 0.05	*p* = 0.1165

Abbreviations: BM_intact_, intact brain metastasis; BM_postop_, post‐operative resection cavity of a brain metastasis; FIP, fast inverse planning; GI, gradient index; MFP, manual forward planning; PCI, Paddick conformity index; Rx IDL, prescription isodose line; sBOT, scaled beam‐on time; TC, target coverage; VS, vestibular schwannoma.

Table 3 lists planner's MFP or FIP plans whose plan quality metrics are statistically significantly different (*p* < 0.05) from those of the clinical plans. Those plans are also marked as pink stars in Figure 2. Junior had the smallest number of MFP plans significantly different from the clinical plans followed by the Senior and Novice in order. This implies that Junior's MFP plans were the most comparable to the clinical plans. Based on plan quality metrics, the Senior's and Novice's MFP plans were superior and inferior to the clinical plans, respectively (Figure 2). A similar trend (i.e., Junior had the smallest number of FIP plans followed by Senior and Novice) was observed for FIP plans (Table 3) but all three planners’ FIP plans were mostly comparable or superior to the clinical plans (Figure 2). The BM_intact_ group had the smallest number of a total of MFP and FIP plans significantly different from the clinical plans.

**TABLE 3:** The results of a paired *t*‐test (parametric) or a Wilcoxon matched pairs test (non‐parametric) between each planner's plans and clinical plans.

	BM_postop_ group	BM_intact_ group	VS group
PCI	N‐MFP, S‐FIP, J‐FIP, N‐FIP	S‐FIP	N‐MFP, N‐FIP
GI	S‐FIP, J‐FIP, N‐FIP	S‐MFP, N‐MFP	S‐FIP, J‐FIP, N‐FIP
No. of shots	S‐MFP, J‐FIP, N‐FIP	None	S‐MFP, N‐MFP
Rx IDL (%)	S‐MFP, N‐MFP	None	N‐MFP
TC (%)	J‐MFP	None	J‐MFP
sBOT (min)	None	J‐MFP, N‐MFP	N‐MFP, N‐FIP
Cochlea D_mean_ (Gy)	N/A	N/A	S‐MFP, S‐FIP
Brainstem D_0.03cc_ (Gy)	N/A	N/A	J‐MFP, N‐MFP

*Note*: Listed plans showed statistically significant difference (*p* < 0.05) when compared with the clinical plans.

Abbreviations: BM_intact_, intact brain metastasis; BM_postop_, post‐operative resection cavity of a brain metastasis; FIP, fast inverse planning; GI, gradient index; J, Junior; MFP, manual forward planning; N: Novice; PCI, Paddick conformity index; Rx IDL, prescription isodose line; S, Senior; sBOT, scaled beam‐on time; TC, target coverage; VS, vestibular schwannoma

### 3.2 FIP parameter settings

**FIGURE 3:**
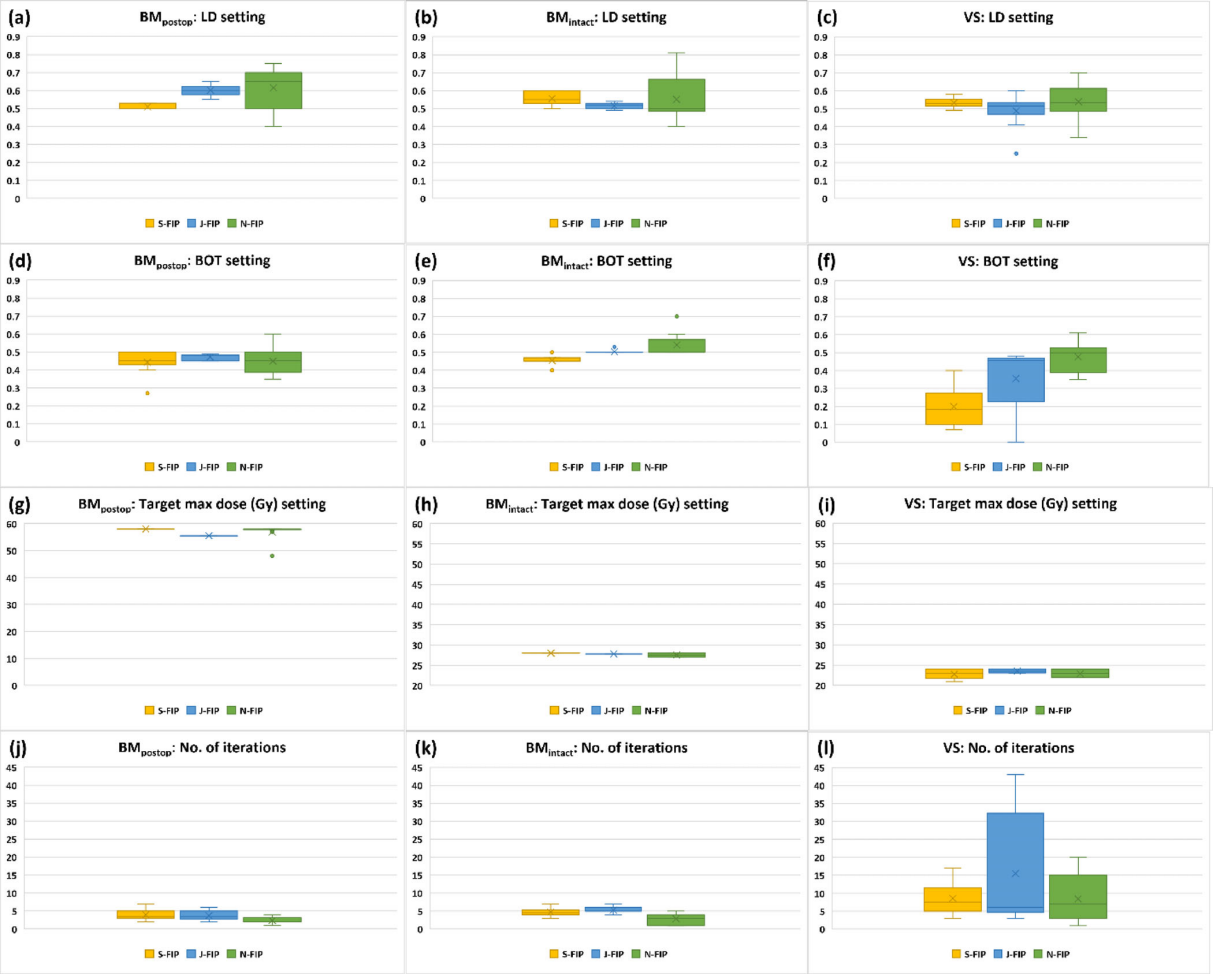
Comparison of FIP parameter settings and number of iterations among three planners. Abbreviations: BM_intact_, intact brain metastasis; BM_postop_, post‐operative resection cavity of a brain metastasis; BOT, beam‐on‐time; FIP, fast inverse planning; J, Junior; LD, low dose; N, Novice; S, Senior; VS, vestibular schwannoma.

Figure 3 shows the comparison of FIP parameter settings and number of iterations among the three planners for all three groups. Table 4 presents the mean values of maximum differences in FIP parameter settings and number of iterations among the planners and the results of statistical analysis. The LD setting was statistically significantly different (*p* < 0.05) among the planners only for the BM_postop_ group (Table 4). For the BM_postop_ group, the mean LD setting was high for Novice, Junior, and Senior in order (Figure 3[a]). The BOT setting was statistically significantly different (*p* < 0.05) among the planners for the BM_intact_ and VS groups (Table 4). In both groups, the mean BOT setting was high for Novice, Junior, and Senior in order (Figure 3[e,f]). The target max dose setting and number of iterations were statistically significantly different (*p* < 0.05) among the planners for the BM_postop_ and BM_intact_ groups but not for the VS group (Table 4). Among the three groups, the VS group had the widest ranges of BOT settings, target max dose settings and number of iterations for all the planners (Figure 3[f,i,l]) and had the greatest mean values of maximum differences in the BOT setting and number of iterations among the planners (Table 4).

**TABLE 4:** Mean values of maximum differences in FIP parameter settings among three planners and the results of a one‐way ANOVA test (parametric) or a Kruskal–Wallis test (non‐parametric) among the planners.

	BM_postop_ group		BM_intact_ group		VS group	
	Mean of max diff. among three planners	ANOVA/Kruskal–Wallis test among three planners	Mean of max diff. among three planners	ANOVA/Kruskal–Wallis test among three planners	Mean of max diff. among three planners	ANOVA/Kruskal–Wallis test among three planners
LD	0.15 ± 0.06 (0.05–0.25)	*p* < 0.05	0.12 ± 0.08 (0.03–0.28)	*p* = 0.4555	0.12 ± 0.09 (0.03–0.33)	*p* = 0.3409
BOT	0.10 ± 0.06 (0.05–0.21)	*p* = 0.5379	0.09 ± 0.08 (0.00–0.30)	*p* < 0.05	0.31 ± 0.15 (0.06–0.51)	*p* < 0.05
Target max dose (Gy)	3.3 ± 2.4 (2.5–10.0)	*p* < 0.05	0.60 ± 0.42 (0.2–1.0)	*p* < 0.05	1.14 ± 0.88 (0.0–2.1)	*p* = 0.1655
No. of iterations	2.4 ± 1.3 (0–5)	*p* < 0.05	3.0 ± 1.8 (1–6)	*p* < 0.05	11.5 ± 11.1 (2–33)	*p* = 0.2072

Abbreviations: BM_intact_, intact brain metastasis; BM_postop_, post‐operative resection cavity of brain metastasis; BOT, beam‐on‐time; FIP, fast inverse planning; LD, low dose; No. of iterations, number of times the FIP optimizer was run; VS, vestibular schwannoma.

### 3.3 Planning time

**FIGURE 4:**
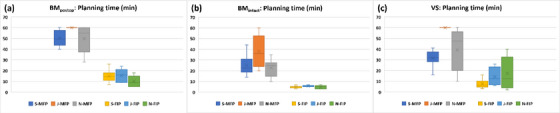
Planning time comparison of MFP or FIP plans among three planners. Abbreviations: BM_intact_, intact brain metastasis; BM_postop_, post‐operative resection cavity of a brain metastasis; FIP, fast inverse planning; J, Junior; MFP, manual forward planning; N, Novice; S, Senior; VS, vestibular schwannoma.

Figure 4 shows the comparison of planning times that the three planners spent on MFP and FIP plans for each group. As expected, mean planning times for MFP plans were longer than those for FIP plans for all the planners in all three groups. For all three groups, planning times for MFP plans were statistically significantly different (*p* < 0.05) among the planners (Table 2). Mean planning times that Senior and Novice spent on MFP plans were longest for the BM_postop_ group and shortest for the BM_intact_ group (Figure 4[a,b]). Junior spent 60 min on all MFP plans for the BM_postop_ and VS groups but less time for the BM_intact_ group (Figure 4). In FIP plans, the mean values of maximum differences in planning times among the planners were smaller and as a result, planning time among the planners was not significantly different (*p* > 0.05) for all three groups (Table 2). Mean planning time that Novice spent on FIP plans was longest for the VS group followed by the BM_postop_ and BM_intact_ groups, whereas the corresponding order for Senior and Junior was BM_postop_, VS and BM_intact_ groups (Figure 4). The VS group had the greatest mean values of maximum differences in planning time among the planners for both MFP and FIP plans (Table 2).

## 4 DISCUSSION

### 4.1 Plan quality metrics

**FIGURE 5:**
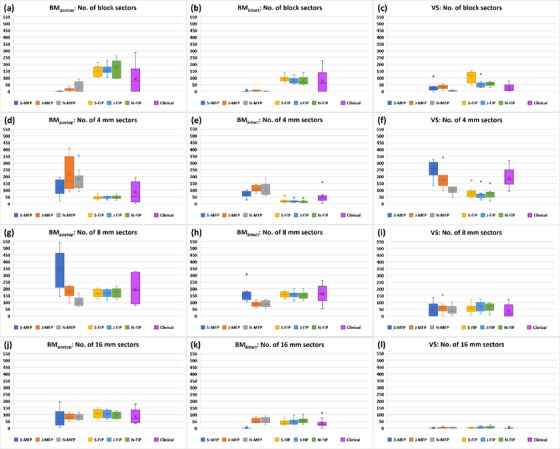
Comparison of sector configurations of MFP or FIP plans among three planners. Abbreviations: BM_intact_, intact brain metastasis; BM_postop_, post‐operative resection cavity of a brain metastasis; FIP, fast inverse planning; J, Junior; MFP, manual forward planning; N, Novice; S, Senior; VS, vestibular schwannoma.

The results in Table 2 demonstrate that inter‐planner plan quality variability for FIP plans was less than for MFP plans. For the BM_intact_ group, the greater mean value of maximum differences in TC of FIP plans among the planners may be attributed to the FIP optimizer's achieving TC between 99.5% and 99.8% (Figure 2[n]). In comparison, TC of MFP plans was achieved mostly up to 99.6% by the planners (Figure 2[n]). For the VS group, the greater mean value of maximum differences in sBOT of FIP plans among the planners is due to the longest sBOT (mean: 58.3 min) obtained by Senior (Figure 2[r]). In our clinic, VS is usually treated with a GK frame and thus, BOT longer than 60 min is acceptable if plan quality can be improved. Senior with experience of more than 15 VS clinical plans, based on clinical experience, achieved the highest PCI (mean: 0.81) for FIP plans by compromising the BOT penalty (mean: 0.2) resulting in more shots (mean: 30.7) and more block and 4 mm collimators (mean: 107.4 [block]; 80.2 [4 mm]) (Figures 2, 3, and 5[c,f]). On the other hand, Junior and Novice with little or no experience of VS clinical plans did not compromise the BOT penalty (mean: 0.36 [Junior]; 0.48 [Novice]) as much as Senior (Figure 3[f]). The mean value of maximum differences in cochlea mean dose of FIP plans among the planners was slightly higher but not much different from that of MFP plans (0.4 ± 0.4 Gy for FIP plans vs. 0.3 ± 0.2 Gy for MFP plans) (Table 2).

**FIGURE 6:**
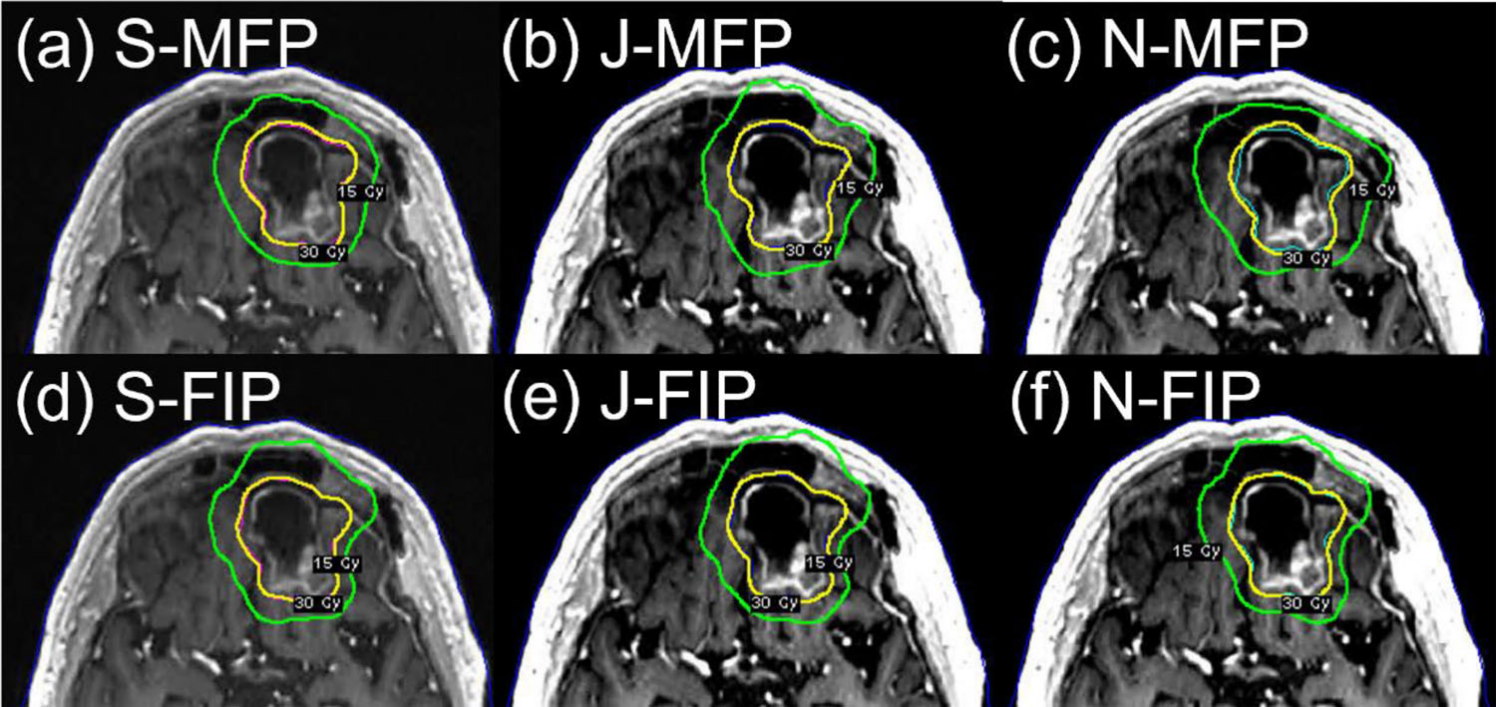
Axial views of representative examples of MFP plans (a–c) and FIP plans (d–f) from the BM_postop_ group generated by three planners showing 100% Rx IDL (30 Gy) and 50% Rx IDL (15 Gy). Abbreviations: BM_postop_, post‐operative resection cavity of a brain metastasis; FIP, fast inverse planning; J, Junior; MFP, manual forward planning; N, Novice; Rx IDL, prescription isodose line; S, Senior.

**FIGURE 7:**
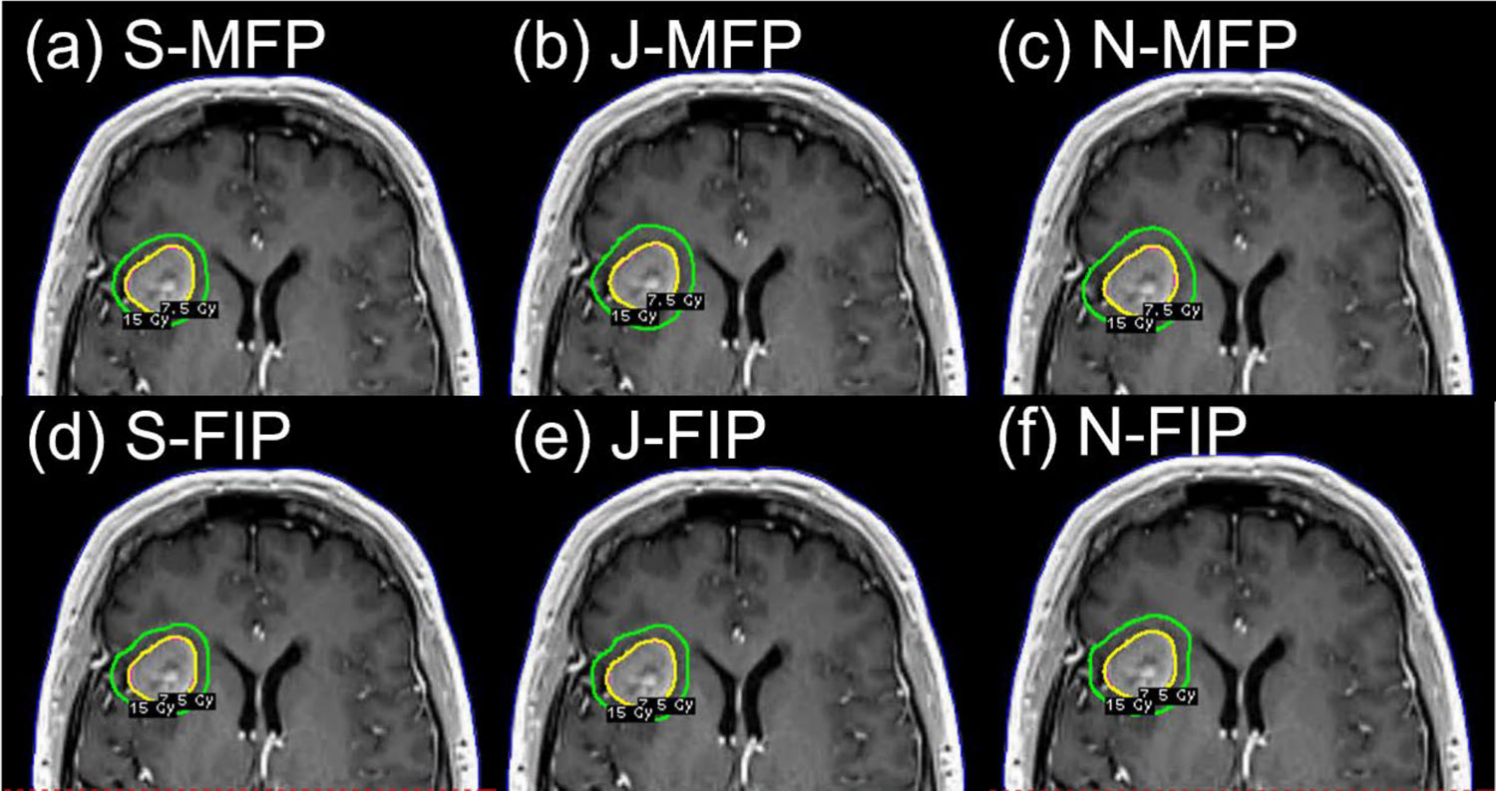
Axial views of representative examples of MFP plans (a–c) and FIP plans (d–f) from the BM_intact_ group generated by three planners showing 100% Rx IDL (15 Gy) and 50% Rx IDL (7.5 Gy). Abbreviations: BM_intact_, intact brain metastasis; FIP, fast inverse planning; J, Junior; MFP, manual forward planning; N, Novice; Rx IDL, prescription isodose line; S, Senior.

**FIGURE 8:**
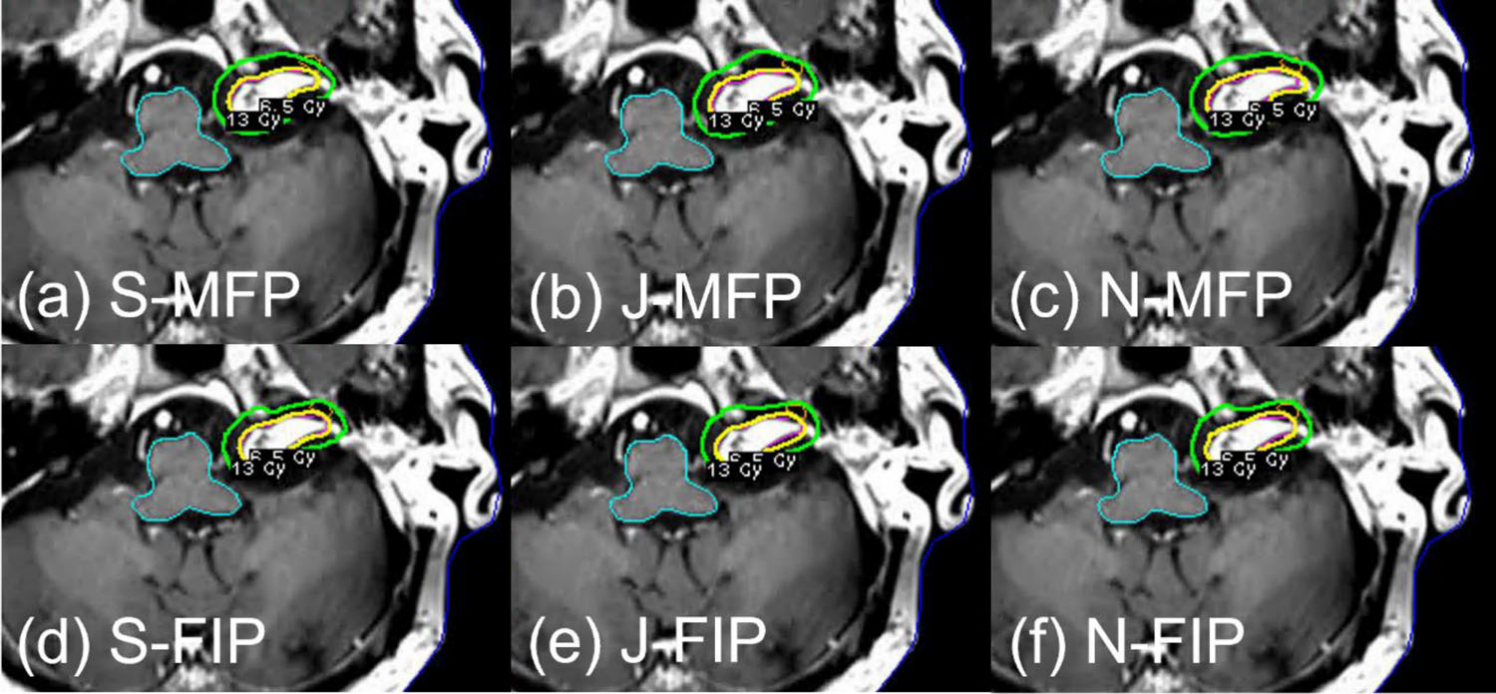
Axial views of representative examples of MFP plans (a–c) and FIP plans (d–f) from the VS group generated by three planners showing 100% Rx IDL (13 Gy) and 50% Rx IDL (6.5 Gy). Abbreviations: FIP, fast inverse planning; J, Junior; MFP, manual forward planning; N, Novice; Rx IDL, prescription isodose line; S, Senior; VS, vestibular schwannoma.

The results of statistical analysis also support less inter‐planner plan quality variability for FIP plans in all three groups (Table 2). For the BM_postop_ group, PCI and number of shots of MFP plans were statistically significantly different among the three planners because Novice achieved much lower PCI (mean: 0.85) and less number of shots (mean: 50) than Senior (mean PCI: 0.91; mean number of shots: 68.1) or Junior (mean PCI: 0.90; mean number of shots: 61.4) (Figure 2[a,g]). To achieve the highest PCI, Senior used more shots, more 8 mm collimators and less 16 mm collimators than Junior or Novice (Figures 2[a,g] and 5[g,j]). Despite some variations in FIP parameter settings among the planners, on the other hand, the differences in most plan quality metrics of FIP plans among the planners were not significant (Table 2 and Figure 3). This may be attributed to relatively similar sector configurations of FIP plans among the three planners (Figure 5). As shown in Figure 6(d–f), FIP plans generated by the planners are fairly similar. For the BM_intact_ group, Junior and Novice achieved plan quality metrics of MFP plans comparable to those achieved by Senior except GI and sBOT (statistically significant differences among the planners) (Table 2). This is because GI and sBOT of MFP plans achieved by Senior were a lot lower and longer than those by Junior or Novice, respectively (mean GI: 2.47 [Senior]; 2.80 [Junior]; 2.90 [Novice] and mean sBOT: 49.1 min [Senior]; 29.9 min [Junior]; 31.9 min [Novice]) (Figure 2[e,q]). Unlike VS cases, in our clinic, metastatic lesions belonging to the BM_intact_ group are usually treated with a GK mask and BOT up to 60 min is allowed if patient can tolerate. For this reason, Senior used more 8 mm collimators and a lot less 16 mm collimators to achieve low GI by compromising BOT than Junior or Novice (Figures 2, 3, and 5[h,k]). Senior with experience > 5 years outperformed in achieving the lowest GI for MFP plans in the BM_intact_ group because of the lesion shape and less complexity of planning (Figure 7[a]). Like for the BM_postop_ group, comparable FIP plans among the planners were generated for the BM_intact_ group as shown in Figure 7(d–f). For the VS group, more plan quality metrics (PCI, GI, number of shots, prescription IDL, and TC) of MFP plans were statistically significantly different among the planners (Table 2). Planner’ level of experience seemed to impact plan quality metrics for the VS group more than for the BM_postop_ and BM_intact_ groups due to the shape and/or size of the lesions as well as proximity to OARs. All the plan quality metrics of FIP plans except sBOT were not significantly different among the planners even with the variations in FIP parameter settings among the planners because of relatively similar sector configurations of FIP plans among the planners (Table 2 and Figures 2, 3, and 5[c,f,i,l]). Representative examples of MFP and FIP plans for the VC group are shown in Figure 8. Regardless of the results of statistical analysis, the differences in TC and prescription IDL of MFP or FIP plans among the planners were relatively small for all three groups (Table 2) because of given institutional GK planning criteria. In addition, for the VS group, cochlea mean dose and brainstem max dose of MFP or FIP plans were not statistically significantly different among the planners (Table 2) because OAR dose constraints were also given to the planners and the planners made efforts to achieve the constraints during planning.

The results in Table 3 indicate that Junior's plans were the most comparable to the clinical plans. Junior is in an intermediate level of GK planning. The clinical plans were generated by multiple planners with varying experience levels. Also, the participating experienced planner(s) (e.g., Senior) who generated some of the clinical plans have gained planning expertise over time and could generate superior plans now to before. For these reasons, Junior's MFP/FIP plans were least frequently seen in Table 3 followed by Senior's and Novice's MFP/FIP plans in order. Due to lack of experience, for the BM_postop_ and VS groups, Novice generated MFP plans inferior to the clinical plans in terms of PCI (Table 3). For the BM_postop_ group, mean PCI of FIP plans was higher than that of the clinical plans for all the planners because of a greater number of shots and different combinations of the four collimators used in FIP plans (Figures 2[a,g] and 5[a,d,g,j]). For the BM_intact_ group, Senior used the lowest BOT (mean: 0.45) and highest LD (mean: 0.56) settings among the planners, resulting in higher PCI (mean: 0.94) for FIP plans than PCI (mean: 0.93) for the clinical plans (Figures 2[b] and 3[b,e]). In FIP plans for the VS group, Novice did not compromise the BOT penalty a lot (mean: 0.48), consequently, leading to lower PCI (mean: 0.78) than PCI (mean: 0.80) for the clinical plans (Figures 2[c] and 3[f]). The FIP optimizer outperformed for GI when the lesions were irregular (BM_postop_ and VS groups) (Figure 2[d,f]). Six clinical plans in the BM_postop_ group and only one clinical plan in the VS group were generated by a combination of FIP and MFP. Therefore, GI of FIP plans generated by all three planners was lower than GI of the clinical plans for the BM_postop_ and VS groups. For the BM_intact_ group, Senior and Novice outperformed and underperformed for GI in MFP plans, respectively, compared with the clinical plans due to their experience level. Senior used the greatest number of shots in MFP plans for the BM_postop_ and VS groups, whereas Novice used the smallest number of shots in MFP plans for the VS group (Figure 2[g,i]). Junior and Novice also used more shots in FIP plans for the BM_postop_ group (Figure 2[g]). This led to the significant differences in the number of shots between these plans and the clinical plans (Table 3). Junior and Novice used a lot less block collimators and more 16 mm collimators in MFP plans than in the clinical plans for the BM_intact_ group (Figure 5[b,k]). Similarly, Novice did not use block collimators much in MFP plans for the VS group (Figure 5[c]). Consequently, sBOT for those MFP plans was shorter and significantly different from sBOT for the clinical plans (Table 3). Novice did not compromise the BOT penalty (mean: 0.48) much in FIP plans for the VS group and as a result, sBOT (mean: 38.9 min) of FIP plans was shorter than that (mean: 50.9 min) of the clinical plans and the difference between the two was significant (Figures 2[r] and 3[f], and Table 3). The differences in cochlea mean dose between Senior's plans and the clinical plans were significant because Senior achieved lower cochlea mean dose in both MFP and FIP plans than in the clinical plans while being able to achieve comparable or better plan quality metrics (Figure 2[s] and Table 3). Junior and Novice achieved higher brainstem max dose in MFP plans than in the clinical plans because they seemed to focus more on achieving cochlea dose constraint as brainstem dose constraint was relatively loose (D_0.03cc_ of 12.5 Gy) considering prescription dose (12.5 or 13 Gy) (Figure 2[t]). Spaniol et al. concluded that the FIP optimizer generated improved plans with reduced BOT compared with clinical MFP plans.^5^ Cui et al. concluded that the FIP optimizer produced equivalent plans with similar BOT compared with clinical MFP plans.^9^ Their findings are similar to our findings demonstrating that all three planners’ FIP plans are comparable or superior to the clinical plans. Due to planners’ experience level, however, in this study, Senior, Junior, and Novice's MFP plans were superior, comparable, and inferior to the clinical plans, respectively.

**FIGURE 9:**
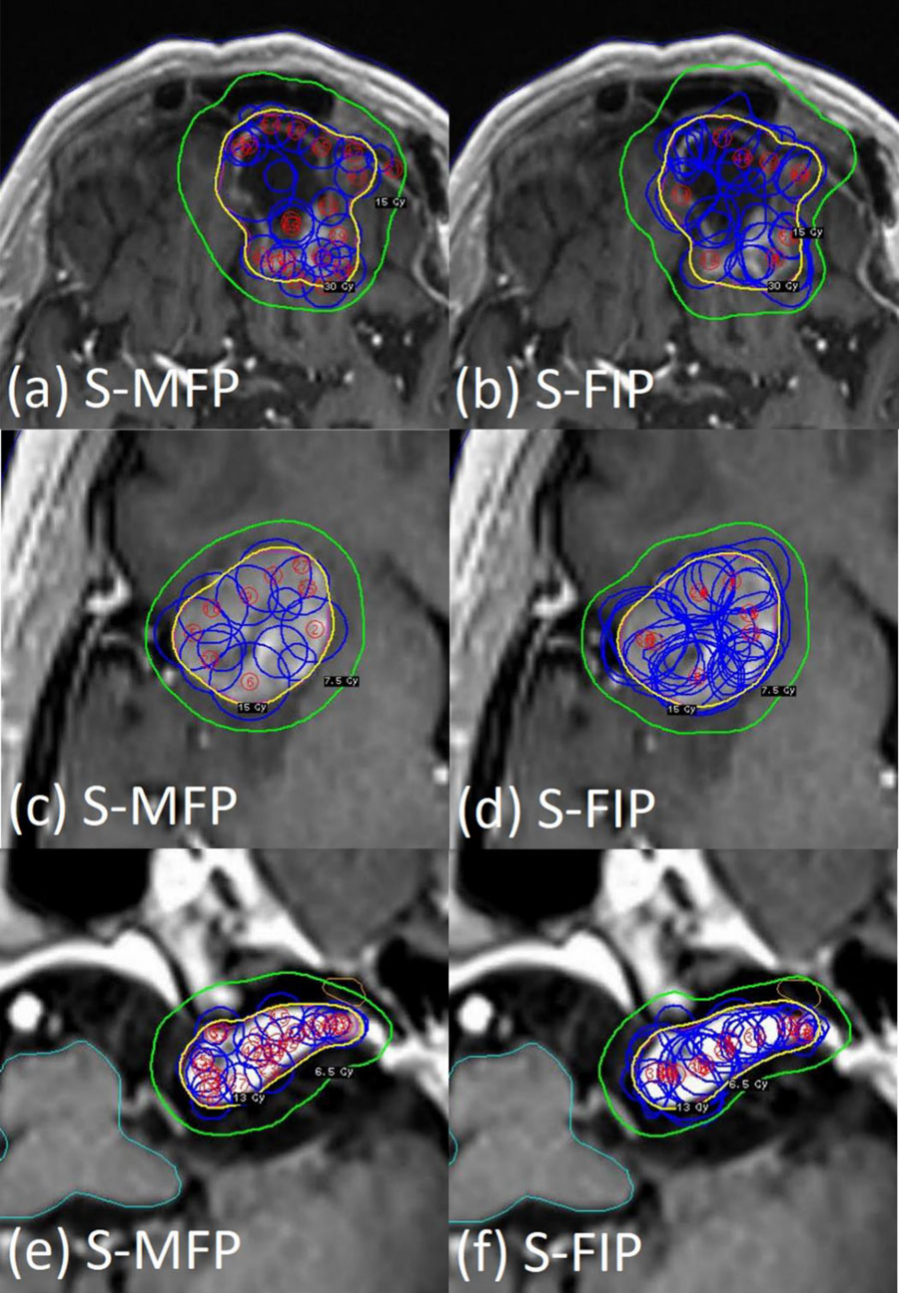
Axial views of representative examples of MFP plans (a), (c), (e) and FIP plans (b), (d), (f) from each group generated by Senior showing beam shapes of shots. Abbreviations: FIP, fast inverse planning; MFP, manual forward planning; S, Senior.

In this study, FIP plans achieved lower GI than MFP plans for all three groups except MFP plans generated by the Senior for the BM_intact_ (Figure 2[d–f]). This result is consistent with the findings of Spaniol et al., Wieczorek et al., and Cui et al.^5^, ^8^, ^9^ The FIP optimizer tends to use various combinations of the collimators.^8^, ^9^ The FIP optimizer optimizes such that shots are well distributed, shots have various beam shapes by combinations of the collimators and notably, multiple shots with different beam shapes are placed at the same isocenter (reusing isocenter) to achieve high PCI, low GI, and short BOT for lesions.^1^, ^8^ For large, irregular lesions (BM_postop_ group), all four collimators were used, and varying beam shapes for shots were possible to achieve lower GI in FIP plans (Table 5 and Figure 9[b]). In contrast, the planner (Senior) did not use combinations of the collimators as effectively as the FIP optimizer and could not find better solutions in MFP plans (Table 5 and Figure 9[a]). Similarly, for small, irregular lesions (VS group), the FIP optimizer mainly used three collimators except the 16 mm collimator due to the lesion size and dose distributions by the combinations of three collimators resulted in lower GI than that in MFP plans (Table 5 and Figure 9[e,f]). For a similar reason, the Junior and Novice did not find better solutions in their MFP plans for all three groups including medium‐sized, spherical lesions (BM_intact_ group) (Figure 2[d‐f]). However, exceptionally, the Senior achieved lower GI in MFP plans than in FIP plans for the BM_intact_ group by predominantly using the 8 mm collimator (Table 5). The 8 mm collimator fit well the curvature of the medium sized, spherical lesions and composite low dose spillage was reduced as shown in Figure 9(c). Senior used the same planning technique for the BM_intact_ group and eight of 10 MFP plans generated by Senior had lower GI than FIP plans (Figure 2[e]). When the shape becomes irregular and size becomes larger or smaller, varying combinations of collimators are required, and the FIP optimizer would outperform the planner (Senior) especially in GI as shown in this study.

**TABLE 5:** Collimator size distributions of each sector in one representative MFP plan and FIP plan from each patient group.

	BM_postop_							
	MFP plan (83 shots; PCI 0.90; GI 2.59)				FIP plan (62 shots; PCI 0.91; GI 2.52)			
Sec. No.	B	4	8	16	B	4	8	16
1	0	6	68	3	12	2	32	16
2	0	8	66	3	17	2	26	17
3	0	6	69	2	12	9	24	17
4	0	7	68	2	22	12	19	9
5	0	7	68	2	19	7	26	10
6	0	8	66	3	29	9	22	2
7	0	7	65	5	21	7	26	8
8	0	5	70	2	18	3	30	11

	BM_intact_							
	MFP plan (42 shots; PCI 0.95; GI 2.39)				FIP plan (49 shots; PCI 0.96; GI 2.64)			
Sec. No.	B	4	8	16	B	4	8	16
1	0	3	38	1	8	2	21	18
2	0	2	40	0	20	1	25	3
3	0	4	38	0	18	2	16	13
4	0	2	40	0	13	2	23	11
5	0	2	40	0	18	1	20	10
6	0	5	37	0	14	2	21	12
7	0	6	36	0	33	0	13	3
8	0	3	39	0	16	0	22	11

	VS							
	MFP plan (43 shots; PCI 0.86; GI 2.74)				FIP plan (40 shots; PCI 0.85; GI 2.51)			
Sec. No.	B	4	8	16	B	4	8	16
1	3	39	1	0	23	15	2	0
2	3	40	0	0	12	17	11	0
3	0	43	0	0	18	15	7	0
4	2	41	0	0	23	10	7	0
5	3	40	0	0	25	12	3	0
6	2	41	0	0	16	11	13	0
7	0	43	0	0	8	17	15	0
8	2	41	0	0	26	10	4	0

*Note*: The plans were generated by the Senior.

Abbreviations: BM_intact_, intact brain metastasis; BM_postop_, post‐operative resection cavity of brain metastasis; FIP, fast inverse planning; GI, gradient index; MFP, manual forward planning; PCI, Paddick conformity index; VS, vestibular schwannoma.

Although this study showed that plan quality metrics for the FIP plans generated without manual adjustments were comparable or superior to those for the clinical plans, in our clinical practice, manual fine adjustments (i.e., moving isocenter of shot(s), changing sector configurations of shot(s) or adding shot(s)) are often preferred to improve TC, PCI, and/or to reduce OAR dose(s). For the 30 clinical plans selected for this study, one patient (3.3%) was planned using the FIP optimizer only, 12 patients (40%) were planned using the FIP optimizer followed by manual adjustments and the rest (17 patients, 56.7%) using MFP. In Wieczorek et al.’s study, 52% (60/115) of the lesions planned using the FIP optimizer required minor manual adjustments to achieve TC comparable to that for clinical MFP plans.^8^

### 4.2 FIP parameter settings

Despite less inter‐planner plan quality variability for FIP plans, some variations in FIP parameter settings among the three planners were observed (Figure 3 and Table 4). For the BM_postop_ group, the LD setting was statistically significantly different among the planners because Junior and Novice used higher LD settings (mean: 0.60 [Junior]; 0.62 [Novice]) than Senior (0.51) to increase PCI (Figure 3[a]). Novice used the highest LD setting (mean: 0.62) and the low BOT setting (mean: 0.45), resulting in high PCI (mean: 0.92) (Figures 2[a] and 3[a,d]). On the other hand, Senior used the lowest LD setting (mean: 0.51) and the lowest BOT setting (mean: 0.44) but not too low LD/BOT settings not to compromise sBOT (around 30 min per fraction) based on clinical practice (Figures 2[p] and 3[a,d]). For the BM_intact_ and VS groups, the BOT setting was statistically significantly different among the planners because Senior used a lower BOT setting than Junior or Novice to increase PCI and to decrease GI (Figures 2[b,c,e,f] and 3[e,f]). Despite statistically significant differences in the target max dose setting and number of iterations among the planners for BM_postop_ and BM_intact_ groups, the absolute differences were not very different (≤ 3.2 Gy for target max dose and ≤ 3 for number of iterations) (Table 4). All the planners had more variations in the BOT setting, target max dose setting and number of iterations for the VS group (Figure 3[f,i,l]) due to the size, shape, and proximity to the OAR(s). Often the 4 mm collimator is big for narrow part of VS lesions. Selecting optimal FIP parameter settings can be challenging and multiple iterations are required to achieve acceptable plan quality metrics. Although plan quality metrics of FIP plans among the planners were not statistically significantly different (Table 2), Senior achieved the highest PCI and the lowest GI for the BM_intact_ and VS groups (Figure 2[b,c,e,f]). From clinical experience, Senior has learned FIP parameter settings which would work well for each group. In other words, clinical experience can improve plan quality for FIP plans. For the VS group, the OAR max dose setting could also impact plan quality metrics. In this study, six lesions in the VS group were adjacent to cochlea and only for those lesions, the OAR max dose was entered in the setting by all the planners. For the same reason, not all the planners entered the OAR max dose for brainstem (data not shown).

### 4.3 Planning time

FIP allowed for shorter planning time and less variability in planning time among the planners for all three groups (Table 2 and Figure 4). In MFP plans, the level of planning experience made planning time significantly different among the planners (Table 2). The differences were more distinct between Senior and Junior because Senior achieved comparable or superior plans to the clinical plans before the time limit, but Junior spent 60 min except for the BM_intact_ group. Novice spent less time on MFP plans than Senior or Junior for the BM_postop_ and BM_intact_ groups (Figure 4[a,b]). Although planning time for FIP plans was shorter, and variability was less and not statistically significantly different among the planners, the level of planning experience still impacted planning time (i.e., Senior spent shorter time on FIP plans than Junior or Novice) (Table 2 and Figure 4).

Planning time had some dependance on the volume and irregularity of the lesion as well as proximity to OAR(s). The volume of the lesions in the BM_postop_ group was largest among the three groups (Table 1) and therefore, planning time for MFP plans was longest for Senior and Novice (mean: 50.4 min [Senior]; 49.8 min [Novice]) (Figure 4[a]). The lesions in the VS group were small but irregular and adjacent to the cochlea/brainstem and thus, planning time for MFP plans was the second longest for Senior and Novice (mean: 31.6 min [Senior]; 39.2 min [Novice]) (Figure 4[c]) but the variability in planning time among the planners was more pronounced (Table 2). All the planners spent the shortest time on MFP plans for the BM_intact_ group because of medium size and spherical shape of the lesions (Figure 4[b]). For FIP plans, running the FIP optimizer took longer for larger lesions (BM_postop_ group, > 1 min/iteration) than for smaller lesions (BM_intact_ and VS groups, < 20 s/iteration). However, Novice spent longer planning time on FIP plans for the VS group than for the BM_postop_ group because of more iterations run to achieve planning criteria (OAR doses and TC) for the VS lesions (Figures 3[j,l] and 4[a,c]) and lack of experience in VS plans. For the same reason for MFP plans, all the planners spent the shortest time on FIP plans for the BM_intact_ group. Planning time for the clinical plans was not recorded at the time of planning and was not included in this study.

### 4.4 Comparison with previous studies

Although similar studies have been previously performed and they reported similar results to ours^5^, ^9^, our study provides more comprehensive information on inter‐planner plan quality variability. Cui et al. examined inter‐planner variability only for FIP plans for post‐operative BM, intact BM, VS, and pituitary adenomas (10 patients per group).^9^ In their study, each patient from 40 clinical plans was replanned using the FIP optimizer without manual adjustments by three planners (two experienced planners with at least 5 years of GK planning experience and one novice with < 3 months experience).^9^ Their observation showed no consistent differences between FIP plans generated by experienced and inexperienced planners.^9^ Spaniol et al. examined inter‐planner variability for both MFP and FIP plans but only three patients (one BM, one VS, and one meningioma) were planned manually and using the FIP optimizer by three experienced planners (two planners with LGP + FIP experience and one planer with LGP experience only).^5^ Their analysis showed that all three planners reached similar plan quality for both MFP and FIP plans.^5^ Compared with these two studies, our study is more comprehensive for the following reasons: (1) Our study investigated inter‐planner plan quality variability for both MFP and FIP plans with more datasets (90 MFP plans and 90 FIP plans) compared with Spaniol et al.’s (nine MFP plans and nine FIP plans).^5^ (2) Three planners who had varying planning experience levels (Senior‐Junior‐Novice) participated in the study. In contrast, Cui et al. had only experienced and novice planners.^9^ Spaniol et al. had all experienced planners and consequently, plan quality for MFP plans among three planners were similar unlike in our study.^5^ (3) A comparison of FIP parameter settings among three planners was made in this study. Wieczorek et al. presented LD/BOT settings, but the settings were only for small/punctate BM lesions.^8^ Variations of FIP parameter settings among multiple planners have not been reported in the literature. (4) In this study, planning time was limited to 60 min and recorded to compare among the three planners for both MFP and FIP plans. This information has not been reported before either.

## 5 CONCLUSION

In this study, we demonstrated that inter‐planner plan quality variability for the FIP plans was less than for the MFP plans. All three planners’ FIP plans were comparable or superior to the clinical plans, whereas Senior's, Junior's, and Novice's MFP plans were superior, comparable, and inferior to the clinical plans, respectively. Also, planning time was shorter and variability in planning time among the planners was less for the FIP plans. Nonetheless, this study showed that the level of planning experience could impact FIP parameter settings for FIP plans based on institutional clinical practice, hence, impacting plan quality metrics even to a small degree.

## AUTHOR CONTRIBUTIONS

Yongsook C. Lee designed the work, collected the data, analyzed the data and drafted the manuscript. D Jay Wieczorek designed the work and collected the data. Vibha Chaswal collected the data and contributed to critical reviews of the manuscript. Rupesh Kotecha, Matthew D. Hall, Martin C. Tom, Minesh P. Mehta, Michael W. McDermott, and Alonso N. Gutierrez contributed to critical reviews of the manuscript. Ranjini Tolakanahalli designed the work and contributed to critical reviews of the manuscript.

## CONFLICT OF INTEREST STATEMENT

Rupesh Kotecha: Personal fees from Accuray Inc., Elekta AB, ViewRay Inc., Novocure Inc., Elsevier Inc., Brainlab, Kazia Therapeutics and Castle Biosciences, and institutional research funding from Medtronic Inc., Blue Earth Diagnostics Ltd., Novocure Inc., GT Medical Technologies, AstraZeneca, Exelixis, ViewRay Inc., Brainlab, Cantex Pharmaceuticals, and Kazia Therapeutics; Mathew D. Hall: Live Like Bella Pediatric Cancer Research Initiative, Florida Department of Health Grants 8LA04 and 22L01; Martin C. Tom: Personal fees from ViewRay Inc. and Elsevier Inc.; Minesh P. Mehta: Consulting fees from Karyopharm, Sapience, Zap‐X, Mevion, Xoft, and Kazia Therapeutics and BOD Oncoceutics and Stock in Chimerix; Michael W. McDermott: Consultant for Stryker Co. and Diende Medical; Alonso N. Gutierrez: Honoraria from ViewRay, Inc., Elekta AB and IBA AB

## Data Availability

Research data are not shared.
